# Drinking Slightly Acidic Electrolyzed Water Promotes Growth Performance, Improves Digestion, and Modulates Antioxidant Status in Weaned Piglets

**DOI:** 10.3390/ani16142124

**Published:** 2026-07-08

**Authors:** Zhifang Shi, Zhengyang Shi, Zike Xu, Ruiting Wei, Shuilin Gao, Xiaoxuan Liang, Yifang Zhang, Zhangying Ye, Lei Xi

**Affiliations:** 1 College of Animal Science and Technology, Henan University of Animal Husbandry and Economy, Zhengzhou 450053, China; shizhifang83158@163.com (Z.S.); 17537202360@163.com (Z.S.); 18538675236@163.com (Z.X.); 18603790582@163.com (R.W.); 13353645561@163.com (S.G.); 19639313896@163.com (X.L.); 15324661889@163.com (Y.Z.); 2 Henan Engineering Research Center on Animal Healthy Environment and Intelligent Equipment, Zhengzhou 450046, China; 3 College of Biosystems Engineering and Food Science, Zhejiang University, Hangzhou 310027, China; yzyzju@zju.edu.cn

**Keywords:** slightly acidic electrolyzed water, weaned piglets, digestive enzyme activity, amino acid transporter, antioxidant

## Abstract

This study assessed the effects of adding slightly acidic electrolyzed water (SAEW) to drinking water (0.3 and 0.6 mg/L) for 15 consecutive days on the growth performance, digestive enzyme activity, amino acid transporter gene expression, antioxidant activity, and immune function in weaned piglets. Using a randomized complete block design with pen as the experimental unit, 144 healthy weaned piglets were randomly assigned to three groups with four replicate pens per group according to weight and sex. During the entire experimental period, the average daily gain and average daily feed intake of the SAEW groups were significantly higher than those of the control (CON) group (*p* < 0.05). As compared to the CON group, the levels of α-amylase, β-amylase, lipase, and proteases in the duodenum increased in both SAEW groups (*p* < 0.05). Furthermore, 0.6 mg/L SAEW positively affected the mRNA levels of *CAT1*, *PepT1*, *CAT2*, *EAAC1*, *rBAT*, *b0,+ AT*, and *4F2hc* in the jejunum and duodenum (*p* < 0.05). Regarding immune function, SAEW significantly decreased intestinal secretory immunoglobulin A levels in a dose-dependent manner (*p* < 0.05), while no significant changes were observed in serum immunoglobulin concentrations between the SAEW groups and the CON group. In terms of antioxidant status, SAEW significantly increased superoxide dismutase and glutathione peroxidase activities, but also increased malondialdehyde content in a dose-dependent manner. Despite the stronger promotion of intestinal amino acid transporters under 0.6 mg/L SAEW, this advantage failed to generate extra growth benefits alongside elevated oxidative damage. In conclusion, both tested SAEW concentrations exert positive effects on piglet growth and digestion; comprehensively balancing production benefits and redox safety, 0.3 mg/L SAEW is the optimal drinking water dosage for weaned piglets.

## Simple Summary

Improving the health and growth of weaned piglets is important for efficient livestock production. Slightly acidic electrolyzed water (SAEW) is a mild sanitizing solution that can be added to drinking water. This study found that adding 0.3 mg/L SAEW to drinking water for 15 days increased weight gain, feed intake, digestive enzyme activity, and antioxidant capacity in weaned piglets, with no harmful effects on their overall immunity. Therefore, SAEW offers a simple and effective way to support piglet health after weaning.

## 1. Introduction

The cleanliness of drinking water in pig farms directly determines the gut health and production performance of pigs [1,2]. However, most pig farms only care about the safety of the source of drinking water, while ignoring the re-contamination problem during its use, which is an important reason for the recurrence of epidemic diseases in large-scale pig farms [3]. Disinfection of drinking water can purify it by eliminating pathogenic microorganisms and preventing the occurrence and prevalence of water-borne diseases, thereby solving the “disease from the mouth” problem of livestock and poultry diseases [4]. Currently, pig farms mainly use chemical disinfectants, including chlorine preparations, iodine preparations, and chlorine dioxide, to disinfect drinking water. However, these have several problems, such as high environmental cost and toxicity to animals and breeders [5,6,7]. Their long-term use can leave chemical residues in the environment and cause antimicrobial resistance [6,7,8]. Without affecting the intestinal health and productivity of pigs, the development and application of environmentally friendly drinking water disinfectants that do not generate antimicrobial resistance have become vital for farms that disinfect drinking water for pigs [9,10]. Therefore, alternative options, including the use of SAEW, are being examined in pig production.

SAEW involves electrolyzing sodium chloride (NaCl) solution or diluted hydrochloric acid (HCl) solution in a non-diaphragm electrolytic device to obtain an electrolysis solution (pH 5.0–6.5) and a high available chlorine concentration (ACC) [11,12]. The antimicrobial effects of SAEW conferred by HOCl have been extensively studied and validated [13,14,15]. Its germicidal activity may be caused either by HOCl or −OCl, which inhibits microbial growth-promoting enzymatic activity, damage to DNA and membranes, and membrane transport ability [11,12]. SAEW not only effectively inhibits diverse microorganisms but also has the desirable characteristics of instantaneous high efficiency, convenient preparation, low cost, no pollution, and no residues, thus making SAEW widely applicable in the fields of food disinfection [16,17,18], medical hygiene [18,19,20], and animal husbandry [21,22].

Initially, SAEW was mainly used for disinfecting breeding environments and livestock products in animal husbandry. Soaking or spraying SAEW has been widely employed in an advanced livestock breeding facility [23]. Zang [21] reported that SAEW exhibits equivalent or higher bactericidal activity for shelled eggs compared to AEW and NaClO solutions and is not only effective in reducing or eliminating *Salmonella Enteritidis* (SE) and *Escherichia coli* (*E. coli*) on shelled eggs but also maintains fresh egg quality during storage. Zheng [24] assessed the SAEW-mediated inactivation efficacy of airborne culturable bacteria attached to particulate matter in hen houses, while Liu et al. [25] found that SAEW was an effective alternative to traditional chemical disinfectants (e.g., benzalkonium bromide) for disinfecting hatching eggs under the conditions of 0.5 mL/egg spray volume, 150 mg/L ACC, and 180 s. In recent years, there have also been reports regarding the application of SAEW as an additive in drinking water for animals. Inagaki et al. [26] suggested that SAEW at 5 ppm of total residual chlorine is an effective and safe alternative for sterilizing drinking water in laboratory animal facilities. The addition of 0.3, 0.5, 0.7, and 1.0 mg/L SAEW to the drinking water of broilers can effectively improve production performance, reduce abnormal behaviors [27], enhance immune function, and reduce intestinal *E. coli* and Salmonella populations [28]. Wang [29] showed that supplementing drinking water with SAEW at 0.3 mg/L could improve the normal feces rate of hens by 10%, while attenuating the number of *E. coli* and pH value. Furthermore, SAEW not only inhibited aquatic microorganisms and increased the water sanitation index, but also promoted the intestinal environment, suppressed the growth of *E. coli*, and enhanced the superior strains of lactic acid bacteria by decreasing the intestinal pH value. Weaned piglets suffer severe weaning stress after maternal separation and feed transition, which triggers intestinal impairment, excessive lipid peroxidation and immunosuppression. Conventional chemical water disinfectants may aggravate such adverse changes, while residue-free SAEW is expected to relieve weaning-related disorders. Nevertheless, very few have reported the effects of adding SAEW to the drinking water of weaned piglets and its effect on their intestinal health and performance.

In this study, different concentrations of SAEW were added to the drinking water of weaned piglets to investigate its effects on their performance, intestinal digestive enzymes, and amino acid transporter (AAT) expression. It investigated the feasibility of using SAEW in the drinking water of weaned piglets and explored whether SAEW supplementation can alleviate weaning stress in piglets. We hypothesized that SAEW supplementation would improve growth performance and intestinal health in weaned piglets by enhancing digestive enzyme activity and antioxidant capacity, despite potential oxidative challenges.

## 2. Materials and Methods

### 2.1. Experimental Design

A total of 144 healthy Duroc × Landrace–Yorkshire ternary hybrid weaned piglets (21 days old, weighing 7.20 ± 0.30 kg, 72 males and 72 females) were selected. Using a randomized complete block design based on body weight and sex, the piglets were randomly assigned to three treatment groups with four replicate pens per group and 12 piglets per pen (6 males and 6 females). The piglets in the CON group, group I, and group II received sterilized tap water, SAEW with 0.3 mg/L available chlorine concentration (ACC), and SAEW with 0.6 mg/L ACC, respectively. The treatment period was 15 days. The pen was considered the experimental unit. The piglets were raised in an airtight nursery pig house with a high-bed slatted floor, a constant temperature (25 °C), and a mechanical ventilation system. During the experiment, piglets had free access to water through nipple drinkers and ad libitum feeding, with the detailed dietary nutritional composition provided in Table 1. The piglets were fed according to the farm regulations, with the pig house being cleaned daily. Food intake, body weight, mental state, and general behavior were recorded throughout the experimental period.

### 2.2. Preparation of SAEW

The storage solution of SAEW was prepared using a non-membrane generator to electrolyze 1 g/L of the NaCl solution containing 100 μg/L HCl. The resulting SAEW stock was diluted with sterile deionized water to the final concentrations of 0.3 (low SAEW) and 0.6 mg/L (high SAEW). The ACC of SAEW was determined using the LH-CLO2M digital display chlorine tester (Shanghai Jiahua Test Equipment Co., Ltd., Shanghai, China). The oxidation-reduction potential (ORP) and pH were measured using the MIK-DM2800 electrode-type tester (Shanghai Kangyi Technology Co., Ltd., Shanghai, China). The ACC, ORP, and pH were 0.09 ± 0.00 mg/L, 310 ± 8.00 mV, and 6.89 ± 0.02, respectively, in the CON group. The ACC, ORP and pH were 0.32 ± 0.03 mg/L, 825 ± 6.00 mV, and 6.22 ± 0.03, respectively, in the low SAEW group. The ACC, ORP, and pH were 0.6 ± 0.07 mg/L, 839 ± 7.00 mV and 6.14 ± 0.03, respectively, in the high SAEW group. All water quality parameters were measured daily at 08:00 and 16:00 to ensure stability throughout the experiment.

### 2.3. Growth Performance

The daily feed intake and feed residue of each group were recorded, and the actual feed intake of each group was calculated based on the difference between the previous day’s feed intake and the current day’s residue. The body weight (BW) of each piglet was measured weekly. The average daily gain (ADG), average daily feed intake (ADFI), and ratio of feed to body weight gain (F/G) were determined based on the group feed intake and the individual body weight data.

### 2.4. Sample Collection and Preservation

On day 15 of the experiment period, two piglets were selected from each replicate group, with a total of eight piglets per group (four males and four females). All physiological and molecular indices including blood biochemical indicators, tissue antioxidant enzyme activities, intestinal gene expression, and digestive enzyme activity were determined based on individual piglets, with a single piglet defined as the experimental unit for relevant statistical analyses. The piglets were weighed after fasting for 12 h. The blood specimens (*n* = 24) were obtained through a jugular vein puncture, transferred into 10 mL heparin-free vacuum tubes (Becton Dickinson Vacutainer, Sumter, SC, USA), and then stored on ice for 60 min. After centrifugation (3000× *g*, 10 min), the serum specimens were obtained and stored at −80 °C until further use. Subsequently, the piglets were anesthetized via intramuscular injection of 4% pentobarbital sodium solution at a dosage of 1 mL/5 kg, performed by a veterinarian from the animal hospital. Complete anesthesia was confirmed by the absence of foot reflexes and corneal reflexes. After achieving full anesthesia, the piglets were euthanized by carotid artery bleeding. The liver tissue was separated on an ice plate and then homogenized with cold saline (weight-to-volume ratio = 1:9). After centrifugation (9000× *g*, 10 min, 4 °C), the liver tissue was stored at −80 °C until the analysis of the antioxidant enzyme (AOE) activities. Intestinal tissue samples were collected to determine gene expression of the intestinal AATs. The small intestines were separated from the mesentery and immediately placed on ice, followed by the dissection of the duodenum, ileum, and jejunum. The intestinal segments (3 cm) of the middle regions of the jejunum were excised, followed by rinsing with PBS; they were then transferred into Eppendorf tubes, snap-frozen in liquid nitrogen, and kept at −80 °C for RT-PCR assays. Finally, 0.5 g of chyme was collected from each intestinal segment (duodenum, ileum, and jejunum), placed in an Eppendorf tube, and kept at −80 °C for assessing digestive enzyme activity.

### 2.5. Detection of Digestive Enzyme Activities

The activities of amylase, lipase, and protease were detected using their ELISA kits (48T, Shanghai Changjin Biotechnology, Shanghai, China). First, the sample or standard was added to the micro-ELISA strip-plate. After binding to their specific antibodies, the samples were subsequently incubated with horseradish peroxidase-conjugated anti-antibodies. The tetramethylbenzidine substrate solution was then added to each well, and the resulting absorbance was measured at 450 nm. The amount of enzyme that reduced the absorbance by 0.001 min^−1^ was defined as one unit of enzyme activity. All assays were conducted in triplicate.

### 2.6. RNA Extraction and RT-PCR Assays

Total RNA was extracted from the jejunum tissues (300 mg) using the RNeasy Mini Kit (Qiagen, Dusseldorf, Germany). Then, cDNA was synthesized using the PrimeScript First Strand cDNA Synthesis Kit (Takara, Tokyo, Japan). The Oligo 7.0 software was used to design the primer pairs (Table 2). RT-PCR assays were performed using the Applied Biosystems 7500 RT-PCR System. The RT-PCR reaction conditions were: 95 °C for 1 min, followed by 35 cycles of 95 °C for 5 s and 58 °C for 30 s, and then gradually heated from 60 °C to 95 °C (heating rate = 0.1 °C/s) with continuous fluorescence assessment. A relative standard curve produced by a series of dilutions (1:107 to 1:1) of the amplified products was employed to quantify the mRNA expression of the target genes. The RT-PCR assays were performed in triplicate. Relative mRNA expression was calculated using the 2^−∆∆CT^ method described by Livak and Schmittgen [30], with β-Actin selected as the stable internal reference gene for normalization. Each sample was analyzed in three technical replicates to guarantee data reliability.

### 2.7. AOE Activities in the Serum and Liver

The total antioxidant capacity, along with malondialdehyde (MDA) content, GSH-Px and SOD activities, was detected with commercially available assay kits (Nanjing Jiancheng Bioengineering Institute, Nanjing, China) as described by Liu [31]. The ferric-reducing antioxidant power assay was utilized to assess the total antioxidant capacity. The MDA content, GSH-Px and SOD activities were evaluated with the corresponding substrates provided in their respective assay kits, and finally, their absorbances were measured at 532, 412 and 450 nm, respectively.

### 2.8. Statistical Analysis

The data were collected from three independent experiments, and all the results were presented as mean ± standard deviation (SD). The graphs were created using Prism software (Version 9.0, GraphPad, San Diego, CA, USA). Statistical tests were performed with the SPSS software Version023.0 (SPSS Inc., Chicago, IL, USA). Prior to analysis, the normality of data distribution was tested using the Shapiro–Wilk test, and the homogeneity of variances was verified using Levene’s test. One-way ANOVA was used to compare the differences between the CON and SAEW groups, followed by Duncan’s multiple range test for post hoc comparisons. *p* < 0.05 was deemed statistically significant.

## 3. Results

### 3.1. Effects of SAEW on Growth Performance

The effect of SAEW on piglet performance is shown in Table 3. On day 1 of the experiment period, BW showed no significant difference between the three groups (*p* = 0.820). On days 7 and 15, the BW in the SAEW groups was significantly higher than that in the CON group (*p* < 0.05). Furthermore, the ADG and ADFI of the SAEW groups continuously increased from day 1 to day 15, with an obvious difference compared to those of the CON group (*p* < 0.05). The F/G ratio in the SAEW group showed a declining trend compared to the CON group, although the difference was not statistically significant.

### 3.2. Effects of SAEW on Digestive Enzyme Activities

**Figure 1:**
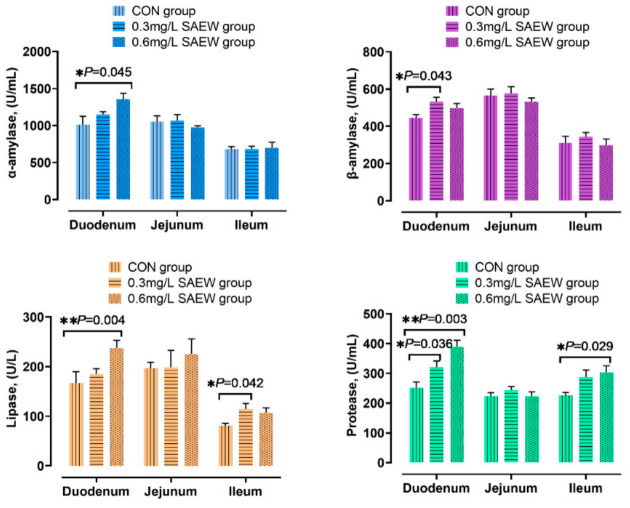
Effects of SAEW on digestive enzyme activity in intestinal chyme (*n* = 8). Note: CON, control; SAEW, slightly acidic electrolyzed water. * indicates significance at the 0.05 level. ** indicates significance at the 0.01 level.

As shown in Figure 1, SAEW significantly promoted the secretion of α-amylase, β-amylase, lipase, and proteases in the duodenum (*p* < 0.05). Although SAEW significantly promoted the secretion of lipase and proteases in the ileum (*p* < 0.05), it did not promote any in the jejunum (*p* > 0.05).

### 3.3. Effects of SAEW on the mRNA Expression of AATs

**Figure 2:**
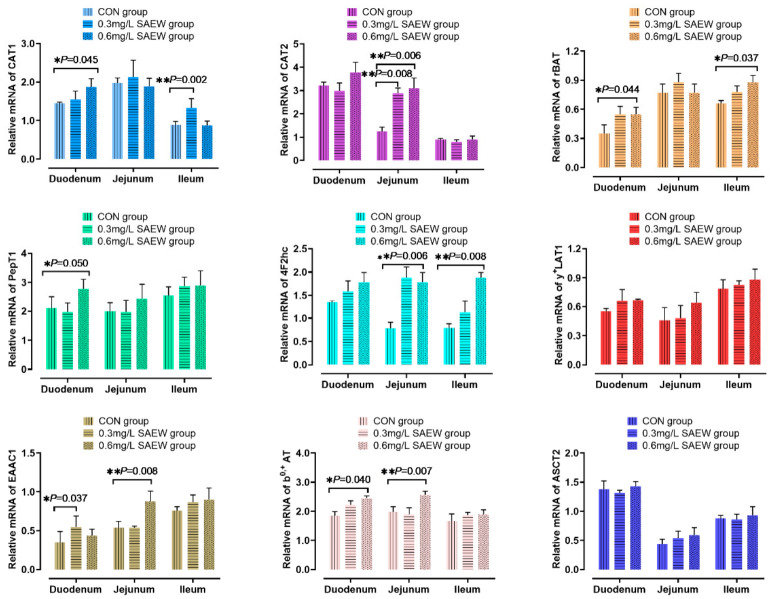
Effects of SAEW on relative mRNA of amino acid transporters (*n* = 8). Note: CON, Control; SAEW, Slightly Acidic Electrolyzed Water; *CAT1*, Cationic Amino Acid Transporter 1; *CAT2*, Cationic Amino Acid Transporter 2; *rBAT*, Related to B0,+ Amino Acid Transporter; *Pep T1*, Peptide Transporter 1; *4F2hc*, 4F2 Heavy Chain; *y + LAT1*, y + L-type Amino Acid Transporter 1; *EAAC1*, Excitatory Amino Acid Carrier 1; *b0,+ AT*, b0,+ -Type Amino Acid Transporter; *ASCT2*, Alanine, Serine, Cysteine Transporter 2. * indicates significance at the 0.05 level. ** indicates significance at the 0.01 level.

The effects of SAEW on the mRNA levels of *AATs* are shown in Figure 2. In the duodenum, the 0.3 mg/L SAEW group showed no significant upregulation of any tested AAT genes (*p* > 0.05). In contrast, the 0.6 mg/L SAEW group significantly increased the mRNA expression of *CAT1*, *CAT2*, *rBAT*, *4F2hc*, *y + LAT1*, *b0,+ AT* and *EAAC1* (*p* < 0.05). In the jejunum, both SAEW concentrations exhibited stimulatory effects. The 0.3 mg/L SAEW group significantly upregulated *CAT2* expression (*p* < 0.05), while the 0.6 mg/L SAEW group comprehensively increased the expression of *CAT1*, *PepT1*, *CAT2*, *EAAC1*, *rBAT*, *b0,+ AT* and *4F2hc* (*p* < 0.05), with particularly pronounced effects on *CAT1*, PepT1 and *CAT2*. In the ileum, the 0.3 mg/L SAEW group only significantly elevated *CAT1* expression (*p* < 0.05), whereas the 0.6 mg/L SAEW group significantly upregulated *CAT1*, *rBAT* and 4F2hc expression (*p* < 0.05).

### 3.4. Effects of SAEW on IgA, IgM, and IgG

The effects of SAEW on IgG, IgM, and IgA concentrations are shown in Table 4. SAEW had no significant effect on the IgG and IgM concentrations in the serum and intestine of piglets (*p* > 0.05). The intestinal IgA concentration in the high SAEW group was significantly lower than that in the control group (CON) and the low SAEW group (*p* < 0.05).

### 3.5. Effect of SAEW on AOE Activities

The effects of SAEW on AOE activities are shown in Table 5, which shows an overall upward trend. Compared with the CON group, SOD activities in the liver and serum were significantly increased in both low and high SAEW groups (*p* < 0.05). Serum SOD in the high SAEW group was further elevated compared with the low SAEW group (*p* < 0.05). For GSH-Px, no significant difference was observed in livers between the CON and low SAEW groups (*p* > 0.05), while the high SAEW group had higher liver GSH-Px activity than the CON group (*p* < 0.05). Meanwhile, serum GSH-Px activities in both SAEW groups were markedly higher than those in the CON group (*p* < 0.05). In addition, liver and serum MDA contents were significantly increased in SAEW groups relative to the CON group (*p* < 0.05), indicating enhanced lipid peroxidation. 

## 4. Discussion

Previous studies demonstrated that the SAEW addition to drinking water can effectively reduce the microbial count in the drinking water pipeline, without adversely affecting the water intake and health status of laying hens, while also promoting the intestinal development of flora in laying hens [32,33]. SAEW supplementation in drinking water can improve broiler performance, immune function, gut morphology, and gut microbial communities [27,28]. Furthermore, with the addition of 3% SAEW, the average daily water and feed intakes of piglets were increased during the first 16 days, and the diarrhea rate was reduced by 100% [34]. However, evidence for SAEW in weaned piglet drinking water remains limited. Here, we investigated the effects of 0.3 and 0.6 mg/L SAEW on growth, digestion, AAT expression, antioxidant status, and immunity.

The study results demonstrated that SAEW had a positive impact on piglet growth performance. Specifically, ADG and ADFI were significantly increased, while the feed-to-weight ratio was markedly reduced. The 0.3 mg/L SAEW treatment significantly improved growth performance, and the 0.6 mg/L SAEW treatment also showed a promoting effect but without further significant improvement compared with the 0.3 mg/L group. This finding is consistent with the results of Hao et al.’s study [34], indicating that SAEW enhances the digestion and absorption of nutrients, thereby promoting piglet weight gain, improving feed conversion efficiency, and enhancing growth performance. This effect may be attributed to alterations in intestinal digestive enzyme activity observed in piglets after SAEW administration.

During the post-weaning period, insufficient production of pancreatic enzymes and gastric acids can limit the absorptive and digestive capacity of the intestinal system [35]. Dietary benzoic acid at 5000 mg/kg was found to promote nutrient digestibility, as well as enhance lipase, amylase, and trypsin activities in the jejunum of weaned piglets [36]. SAEW could improve digestive tract function by elevating digestive enzyme activities in the gastric mucosa, which maximized nutrient absorption and improved performance [27,29]. In this study, SAEW increased the α-amylase, β-amylase, lipase, and protease activities of the intestine compared to the CON group, thereby improving the performance of weaned piglets.

*AATs* are transmembrane channels that mediate amino acid transport across cell membranes and support essential cellular functions. They can be classified into acidic *AATs*, basic *AATs*, and small peptide transporters, with each type exhibiting specific substrate preferences for amino acid absorption. The acidic *AAT EAAC1*, a member of the *EAAT* family, is the most important high-affinity transporter, primarily responsible for glutamate and aspartate uptake in the intestine [37]. Previous studies have shown that *EAAC1* expression in the jejunum of early-weaned piglets is 88% lower than that in suckling piglets due to weaning stress [38]. In this study, the 0.6 mg/L SAEW group had 2.49-fold higher *EAAC1* expression in the jejunum than the CON group, and this level was also significantly higher than that in the 0.3 mg/L SAEW group. This indicates that high-concentration SAEW can significantly upregulate jejunal *EAAC1* expression, thereby promoting glutamate and aspartate transport in the jejunum and alleviating weaning stress in piglets. Consistent with another study showing that intestinal *AAT* levels gradually decrease after weaning [39], we observed low basal *AAT* expression in the control group. Our findings demonstrate that 0.6 mg/L SAEW significantly increases the expression of *CAT1*, *rBAT*, and *4F2hc* in the ileum. These transporters are mainly involved in the absorption of basic and neutral amino acids, indicating that high-concentration SAEW can enhance amino acid absorption in the ileum. Additionally, 0.6 mg/L SAEW significantly upregulated *PepT1* expression in the jejunum, further improving the utilization of dietary protein.

Body cells have developed a complex antioxidant defense system comprising endogenous enzymatic/non-enzymatic antioxidants. These antioxidants can scavenge free radicals, thus reducing their damage to important biomolecules and eventually to body tissues. They can be classified as the first, second, third, and even fourth lines of defense based on their responses against a free radical invasion. The role and effectiveness of the first line of defense antioxidants, including GSH-Px, CAT, and SOD, are vital and indispensable in the entire antioxidant-mediated defense mechanism [40,41]. SOD, one of the most important AOEs, scavenges superoxide anion radicals, enhances immune function, and improves disease resistance. GSH-Px is another essential AOE, which can scavenge lipid peroxides and hydrogen peroxide radicals. This study demonstrated that the addition of SAEW led to an upward trend in the levels of GSH-Px and SOD in the liver and serum. Compared to the control group, high SAEW significantly increased the content of GSH-Px and SOD in the blood and liver. A dose-dependent increase in MDA content was observed in serum and liver as the SAEW concentration increased. As a major end-product of lipid peroxidation, MDA is a classic biomarker of oxidative damage rather than improved antioxidant status. The gradual elevation of MDA with increasing SAEW dosage indicates that SAEW induces mild oxidative stress in a dose-dependent manner. Therefore, SAEW activates the enzymatic antioxidant system in weaned piglets but simultaneously causes mild oxidative stress. The increased SOD and GSH-Px represent a compensatory response to counteract ROS-induced lipid peroxidation. Under the present experimental conditions, 0.3 mg/L SAEW markedly improved growth and digestive function without obvious accumulation of MDA. Although 0.6 mg/L SAEW further boosted antioxidant enzyme activities and evidently upregulated intestinal amino acid transporter expression to facilitate amino acid uptake, this extra antioxidant activation failed to generate further improvements in growth and nutrient absorption, accompanied by aggravated lipid peroxidation; the persistent lipid peroxidation may disrupt enterocyte metabolism and divert energy toward sustaining antioxidant compensation, thereby counterbalancing the growth advantages of enhanced amino acid absorption and resulting in no extra performance gain at 0.6 mg/L SAEW.

Serum and gut immunoglobulins are crucial markers for assessing the immune status of an animal [29]. This study showed that 0.3 and 0.6 mg/L SAEW had no significant effects on IgG and IgM concentrations in both the serum and intestine of weaned piglets, confirming the good systemic immunological safety of low-concentration SAEW, which is consistent with Inagaki’s [26] findings in mice. Notably, the intestinal IgA level in the 0.6 mg/L group was significantly lower than that in the control group and the 0.3 mg/L group. However, the underlying mechanisms remain unclear. Therefore, although decreased IgA levels may indicate local mucosal immunomodulation, their relationship with growth performance requires further investigation and validation.

## 5. Conclusions

Under the conditions of this study, adding 0.3 or 0.6 mg/L of SAEW to drinking water improved the growth performance and digestive enzyme activity of weaned piglets, inducing compensatory upregulation of SOD and GSH-Px. Concurrently, MDA levels increased with rising concentrations, particularly at 0.6 mg/L. Although 0.6 mg/L significantly enhanced the expression of intestinal amino acid transporter genes, it did not translate into superior growth outcomes and was associated with elevated oxidative stress and reduced intestinal immunoglobulin A (IgA) levels. Therefore, considering the balance between therapeutic efficacy and oxidative safety, 0.3 mg/L is recommended as the optimal addition concentration of SAEW in drinking water for weaned piglets.

## Figures and Tables

**Table 1:** Composition and nutrients of the basic diet (air-dried basis).

Item	Content	Item	Content
Composition (%)		Nutrients	
Maize	60.50	Digestible energy (MJ/kg)	14.10
Fish meal	5.00	Crude protein (%)	20.21
Maize protein powder	5.00	Calcium (%)	0.76
Soybean oil	1.00	Available phosphorus (%)	0.40
Soybean meal (crude protein 43%)	24.00	Lysine (%)	1.25
Limestone powder	1.18	Methionine (%)	0.43
Dicalcium phosphate	1.30	Threonine (%)	0.71
L-lysine (98%)	0.60	Tryptophan (%)	0.16
DL-methionine (98%)	0.13		
Threonine	0.17		
Tryptophan	0.02		
Choline chloride	0.10		
Table salt	4.00		
Premix	6.00		

Note: The premix provided the following nutrients for every 1 kg of feed: vitamin A 6000 IU, vitamin D3 400 IU, vitamin E 30 mg, vitamin K3 2 mg, vitamin B1 3.5 mg, vitamin B2 5.5 mg, vitamin B6 3.5 mg, vitamin B12 25.0 μg, biotin 0.05 mg, folic acid 0.3 mg, D-pantothenic acid 20 mg, niacin 20 mg, choline chloride 500 mg, iron 110 mg, zinc 100 mg, copper 20 mg, manganese 40 mg, selenium 0.30 mg, and iodine 0.40 mg. (The nutrient contents are calculated values.).

**Table 2:** Primers used for real-time polymerase chain reaction.

Genes	Primers	Sequences (5′–3′)	Size (bp)	Tm (°C)	Accession No.
*CAT1*	Forward	TGCCCATACTTCCCGTCC	192	59	NM_001012613
	Reverse	GGTCCAGGTTACCGTCAG			
*CAT2*	Forward	TGCCCATACTTCCCGTCCGT	182	60	NM_001012619
	Reverse	GGTCCAGGTTACCGTCAGTC			
*rBAT*	Forward	TTTCCGCAATCCTGATGTTC	146	59	NM_001123042
	Reverse	GGGTCTTATTCACTTGGGTC			
*Pep T1*	Forward	CCCAGGCTTGCTACCCAC	144	60	NM_214347
	Reverse	ACCCGATGCACTTGACGA			
*4F2hc*	Forward	CTCGAACCCACCAAGGAC	174	59	XM_003361818
	Reverse	GAGGTGAGACGGCACAGAG			
*y + LAT1*	Forward	GCCCATTGTCACCATCATC	216	59	NM_001110421
	Reverse	GAGCCCACAAAGAAAAGC			
*EAAC1*	Forward	CACAACAACTGCGAGAAGGA	155	60	DQ231579
	Reverse	CCGTTGATAAGCGTCAGGAT			
*b0,+ AT*	Forward	ATCGGTCTGGCGTTTTAT	144	59	NM_001110171
	Reverse	GGATGTAGCACCCTGTCA			
*ASCT2*	Forward	GCCAGCAAGATTGTGGAGAT	206	60	DQ231578
	Reverse	GAGCTGGATGAGGTTCCAAA			
β-Actin	Forward	TGCGGGACATCAAGGAGAAG	216	60	XM_003357928
	Reverse	AGTTGAAGGTGGTCTCGTGG			

Note: *ASCT2*, Na^+^-neutral AA exchanger; *B0AT1*, system B0 neutral AA transporter; *CAT-1*, cationic amino acid transporter 1; *b0,+ AT*, related to b0,+ amino acid transporter; *y + LAT1*, y + L amino acid transporter-1; *4F2hc*, 4F2 heavy chain; *Pept-1*, intestinal peptide transporter; *rBAT*, basic amino acid transporter; Tm, melting temperature.

**Table 3:** Effects of SAEW on growth performance of piglets (*n* = 4).

Items	CON Group	Low SAEW Group	High SAEW Group	SEM	*p*-Value
BW (kg)					
1 d	7.20 ± 0.04	7.21 ± 0.10	7.23 ± 0.06	0.01	0.820
7 d	10.85 ± 0.60 ^a^	11.62 ± 0.51 ^b^	11.78 ± 0.37 ^b^	0.16	0.029
15 d	15.16 ± 0.67 ^a^	17.46 ± 0.50 ^b^	17.70 ± 0.49 ^b^	0.28	<0.001
ADG (kg)					
1–7 d	0.53 ± 0.08 ^a^	0.63 ± 0.08 ^b^	0.65 ± 0.05 ^b^	0.02	0.045
7–15 d	0.61 ± 0.12 ^a^	0.73 ± 0.05 ^b^	0.75 ± 0.09 ^b^	0.03	0.054
1–15 d	0.57 ± 0.04 ^a^	0.68 ± 0.04 ^b^	0.70 ± 0.04 ^b^	0.02	<0.001
ADFI (kg)					
1–7 d	0.78 ± 0.04 ^a^	0.85 ± 0.05 ^b^	0.88 ± 0.05 ^b^	0.02	0.013
7–15 d	1.00 ± 0.07 ^a^	1.09 ± 0.07 ^b^	1.12 ± 0.08 ^b^	0.02	0.039
1–15 d	0.89 ± 0.03 ^a^	0.98 ± 0.06 ^b^	1.00 ± 0.05 ^b^	0.02	0.006
F/G					
1–7 d	1.50 ± 0.13 ^a^	1.34 ± 0.08 ^b^	1.35 ± 0.07 ^b^	0.03	0.033
7–15 d	1.65 ± 0.11 ^a^	1.48 ± 0.14 ^b^	1.47 ± 0.11 ^b^	0.04	0.048
1–15 d	1.58 ± 0.13 ^a^	1.41 ± 0.11 ^b^	1.42 ± 0.09 ^b^	0.03	0.068

Note: Different lowercase letters in the same row indicate significant differences between groups (*p* < 0.05), while no letter or the same letter indicates insignificant differences (*p* > 0.05). The same notation applies to Table 4 and Table 5. SAEW, slightly acidic electrolyzed water; BW, body weight; ADG, average daily gain; ADFI, average daily feed intake. d means days.

**Table 4:** Effects of SAEW on immunoglobulin IgG, IgM, and IgA concentrations (*n* = 8).

Items	CON Group	Low SAEW Group	High SAEW Group	SEM	*p*-Value
IgG (ng/L)					
intestine	16.80 ± 0.11	16.82 ± 0.24	16.73 ± 0.26	0.13	0.977
serum	21.23 ± 1.44	19.06 ± 1.55	20.70 ± 1.37	1.98	0.750
IgM (ng/L)					
intestine	10.43 ± 0.10	9.52 ± 0.12	10.56 ± 0.88	2.47	0.778
serum	12.63 ± 0.14	11.70 ± 0.99	12.73 ± 1.10	3.11	0.603
IgA (ng/mL)					
intestine	2.36 ± 0.09 ^a^	2.45 ± 0.19 ^a^	1.88 ± 0.17 ^b^	0.13	0.050
serum	1.93 ± 0.15	1.98 ± 0.36	2.02 ± 0.23	0.46	0.665

Note: IgG, immunoglobulin G; IgM, immunoglobulin M; IgA, immunoglobulin A. Different lowercase letters in the same row indicate significant differences between groups (*p* < 0.05), while no letter or the same letter indicates insignificant differences (*p* > 0.05).

**Table 5:** Effects of SAEW on antioxidant enzyme activities (*n* = 8).

Items	CON Group	Low SAEW Group	High SAEW Group	SEM	*p*-Value
SOD (U/mL)					
liver	234.09 ± 30.30 ^a^	313.55 ± 20.32 ^b^	316.87 ± 30.00 ^b^	20.30	0.007
serum	221.23 ± 21.45 ^a^	300.01 ± 18.90 ^b^	340.70 ± 22.56 ^c^	21.98	0.000
GSH-Px (U/mL)					
liver	2.71 ± 0.10 ^a^	2.65 ± 0.42 ^a^	3.09 ± 0.44 ^b^	7.50	0.048
serum	3.05 ± 0.09 ^a^	5.27 ± 0.84 ^b^	5.76 ± 0.50 ^b^	18.44	0.003
MDA (nmol/mL)					
liver	4.24 ± 0.71 ^a^	5.45 ± 0.64 ^b^	5.80 ± 0.69 ^b^	13.66	0.050
serum	7.88 ± 0.12 ^a^	11.55 ± 1.67 ^b^	12.06 ± 1.89 ^b^	4.62	0.005

Note: SOD, superoxide dismutase; GSH-Px, glutathione peroxidase; MDA, malondialdehyde. Different lowercase letters in the same row indicate significant differences between groups (*p* < 0.05), while no letter or the same letter indicates insignificant differences (*p* > 0.05).

## Data Availability

Data will be provided within the manuscript.
